# Surfce Functionalized via AdLAMA3 Multilayer Coating for Re-epithelization Around Titanium Implants

**DOI:** 10.3389/fbioe.2020.00624

**Published:** 2020-06-11

**Authors:** Jing Zhang, Yongzheng Li, Jialu Li, Yuan Shi, Jinxing Hu, Guoli Yang

**Affiliations:** ^1^The Affiliated Stomatology Hospital, Zhejiang University School of Medicine, Hangzhou, China; ^2^Key Laboratory of Oral Biomedical Research of Zhejiang Province, Zhejiang University School of Stomatology, Hangzhou, China

**Keywords:** titanium implant, LAMA3, *in situ* gene transduction, peri-implant epithelium, re-epithelization

## Abstract

The peri-implant epithelium (PIE) forms a crucial seal between the oral environment and the implant surface. Compared with the junctional epithelium (JE), the biological sealing of PIE is fragile, which lacks hemidesmosomes (HDs) and internal basal lamina (extracellular matrix containing laminin332, IBL) on the upper part of the interface. In the study, we aim to prepare a coating with good biocompatibility and ability to immobilize the recombinant adenovirus vector of LAMA3 (AdLAMA3) for promoting the re-epithelization of PIE. The titanium surface functionalized with AdLAMA3 was established via layer-by-layer assembly technique and antibody-antigen specific binding. The biological evaluations including cell adhesion and the re-epithelization of PIE were investigated. The results *in vitro* demonstrated that the AdLAMA3 coating could improve epithelial cell attachment and cell spreading in the early stage. *In vivo* experiments indicated that the AdLAMA3 coating on the implant surface has the potential to accelerate the healing of the PIE, and could promote the expression of laminin α3 and the formation of hemidesmosomes. This study might provide a novel approach and experimental evidence for the precise attachment of LAMA3 to titanium surfaces. The process could improve the re-epithelization of PIE.

## Introduction

Currently, dental implants have become important and popular prosthodontic devices for replacing missing teeth due to caries or periodontal disease in clinic (Hong and Oh, 2017). And the titanium has also been considered the standard material for the treatment of edentulous jaws (Nishihara et al., 2019). Although the use of dental implants to rehabilitate the loss of teeth has markedly increased in the last 30 years (Jenny et al., 2016), peri-implantitis is a significant complication of dental implants that is caused by local factors such as oral bacterial infection, systemic factors such as nutritional status, hormonal abnormality and hematologic disorder (Reynolds, 2014; Holtfreter et al., 2015; Lee et al., 2018). Peri-implantitis is considered to be the major cause of implant failure. For structural reasons, the biological sealing between the implant and adjacent soft tissue is closely related to the initiation of peri-implantitis (Ramanauskaite and Juodzbalys, 2016; Schwarz et al., 2018). Therefore, it is critical to establish a strong biological epithelial sealing at the implant surface in the cervical region to prevent peri-implantitis and increase the survival rate of dental implants. Surface modifications, such as fabrication of a biomimetic antibacterial multilayer coating to prevent implant infection (Ao et al., 2019), implant surface modification with protease activated receptor 4-activating peptide to prevent bacterial attachment and invasion (Maeno et al., 2017), and functionalization with superparamagnetic TiO2 coatings to prevent soft tissue recession and inflammatory reaction (Li et al., 2019), have been widely studied in the area of dental implant materials in recent years. However, no surface modification strategy to date has been able to create a perfect biological sealing structure around the transmucosal implant.

The junctional epithelium (JE) around a tooth is known to connect to the enamel via hemidesmosomes (HDs) and a basal-lamina-like extracellular matrix, called the internal basal lamina (IBL). HDs are one of the most important biological sealing structures for epithelial cells via laminin332, integrin α6β4 (Atsuta et al., 2019). Similarly, it has been reported that the peri-implant epithelium (PIE) can make close contact with the implant surface through these unique structures (Yeung, 2008). Ultrastructural observations have demonstrated that the IBL consists of the lamina densa and the lamina lucida, of which the former connects to the implant surface, while the latter connects to peri-implant epithelial cells. However, Atsuta et al. (2012) reported that HDs and IBL were observed mainly at the apical portion of the interface between the implant and the PIE.

Previously, many studies have demonstrated that laminin332 is an important component in IBL and interacts with integrin α6β4 to form HDs (Yamashita et al., 2010; Koidou et al., 2018). As Langhofer’s study reported, keratinocytes cultured on the extracellular matrix secreted by 804G cells (mainly containing laminin332) were induced to “trigger” hemidesmosome assembly (Langhofer et al., 1993). There is a globular domain (G domain) in the α3 chain of laminin332, which plays a significant role in both the nucleation of HDs and the maintenance of HDs integrity (Yamashita et al., 2010). Moreover, it has been reported that the synthesis of α3 chain was a limiting factor in the process of laminin332 heterotrimer assemblyn (Matsui et al., 1995). Therefore, the G domain of the laminin332 α3 chain was chosen as the functional gene section in this research.

Gene delivery has become an important method for the overexpression of recombinant proteins to avoid the instability and immunogenicity of the therapeutic protein delivery systems. With an appropriate vector, the exogenous gene can be overexpressed shortly by transferring the target gene into the host cell. Compared with non-viral vectors, the adenoviral vectors have been widely used in gene therapy because of the higher transfection efficiency. Although bolus delivery by direct injection is the easiest viral delivery method, the virus may diffuse and induce an immune response (Kim et al., 2016). Integrating non-viral/viral gene delivery with biocompatible materials is a promising reverse transfection strategy to overcome a number of disadvantages, including inefficient delivery, limited tropism, immune responses and spread of vectors to distant sites (Jang et al., 2011; Huang et al., 2015; Rey-Rico et al., 2016; Yang et al., 2016).

In the study, we aim to prepare a coating with good biocompatibility and ability to immobilize the recombinant adenovirus vector of LAMA3 (AdLAMA3). The anti-adenovirus antibody functionalized multilayer coating on the titanium was fabricated according to the method described by Lin et al. (2010), and then the AdLAMA3 could be immobilized on the coating via the specific antibody-antigen binding. The biological evaluations including cell adhesion and the re-epithelization of PIE were investigated.

## Materials and Methods

### Fabrication of LAMA3 Functionalized Multilayer Coatings on Ti

#### Preparation of Titanium Plates

Titanium plates (8 mm^2^ × 8 mm^2^) were polished with abrasive silicon carbide paper of various grain sizes (from #320 to #4000) and then rinsed in an ultrasonic cleaner with acetone, 75% alcohol, and distilled water for 15 min, respectively, and dried in a nitrogen atmosphere.

#### Fabrication of Multilayer Coatings by the Layer-by-Layer Technique

Multilayer coatings were fabricated on polished titanium plates using the layer-by-layer (LBL) technique. Briefly, chitosan (CS) (5 mg/ml) (Yunzhou Biochemistry, Qingdao, China) in acetic acid buffer solution (pH 4.5), and hyaluronic acid (HA) (0.5 mg/ml) (Sigma Chemical Co., MO, United States) in distilled water, and IV collagen solution (COL) (50 μg/ml) (Sigma-Aldrich, St. Louis, MO, United States) in acetic acid buffer solution (pH 4.5) were used sequentially for the LBL assembly. Smooth titanium plates were immersed in the CS solution for 30 min to form a precursor layer with a positive charge, initiating the assembly process. Samples were then immersed into the HA solution for 5 min, and then were dipped into the IV COL solution for 5 min to generate a (HA/COL) double layer, which was referred as CS/(HA/COL)1. Similarly, the multilayer coating was referred as CS/(HA/COL)n after repeating the cycle continuously several times (n), and the coating after chemical cross-linking was referred as CS/(HA/COL)n-CL.

#### Characterization of the CS/(HA/COL)n Coating

The surface of the CS/(HA/COL)n coating was observed by field-emission scanning electron microscopy (FE-SEM, SIRION-100, FEI, United States). Contact angle measurements were carried out using a dynamic contact angle system (SL200B, Solon Tech. Inc., Ltd., Shanghai, China), with ultrapure water as the wetting agent. Each contact angle reported here is the mean of three independent measurements.

#### Detection of Cell Proliferation Activity

HaCaT cells were seeded onto different samples with a density of 2 × 10^4^/well. After 1, 3, and 5-day of seeding, cell proliferation was evaluated using Cell Counting kit-8 (Dojindo, Japan). At the setting time point, culture medium was replaced with 500 μl of fresh medium containing 50 μl of CCK-8 reagent, and the samples were incubated at 37°C for 2 h. The optical densities (OD) of the mixed solution were measured using a SpectraMax microplate reader (Molecular Devices, United States) with wavelengths of 450 nm. Each group contained 3 samples, and the mean value served as the final result.

#### Adenovirus Immobilization on the CS/(HA/COL)n Coatings and Condition Optimization

Recombinant adenovirus encoding LAMA3 (AdLAMA3) was constructed by Hanbio Biotech Company (Shanghai, China). The titanium specimen with CS/(HA/COL)n coating was immersed into 0.25% glutaraldehyde for 2 h for cross-linking and facilitating to immobilize the anti-adenovirus hexon antibody. The 50 μg/well anti-hexon antibody (AbD-Serotec, United States) was introduced onto the modified surface overnight.

The dose of andenovirus immobilized on the coatings was opitimized according to the most effective transfection rate. Briefly, a series of quantities of AdLAMA3 (0.05, 0.1, 0.5, 1, 5 × 10^8^ PFU) were immobilized on titanium surface in 48-well plates, and then HaCaT cells were seeded on the plates at a density of 2 × 10^4^/well. After the gene transfection of 48 h, the transfection rate was detected by fluorescent microscopic observation of green fluorescent protein (GFP) expression.

### Cell Adhesion Assessments

To study the cell adhesion on CS/(HA/COL)5 coating, CS/(HA/COL)5-AdGFP coating, and CS/(HA/COL)5-AdLAMA3 coating and the smooth titanium surface, HaCaT cells were seeded on the 8 mm^2^ × 8 mm^2^ plates at a density of 2 × 10^4^/well in 48-well plates. After 1, 3, and 6 h of incubation, the samples were rinsed with 0.01 M of PBS 3 times to remove the unattached cells, and then fixed by 4% paraformaldehyde at 4°C for 20 min. After being rinsed with PBS, the samples were incubated with 0.5% bovine serum albumin for 30 min to prevent non-specific binding. The cell actin microfilaments were then stained using rhodamine labeled phalloidin (Cytoskeleton Inc., United States) at a 1:40 dilution. After being rinsed three times with PBS, nuclei were stained with Hoechst 33258 DNA dye (Beyotime, China) for 5 min. Finally, the samples were observed and photographed with a fluorescence microscope (Eclipse-80i; Nikon, Tokyo, Japan). The cell number was measured in 10 randomly selected areas on each sample using Image-Pro version 6.0 software (Media Cybernetics Corp., United States), three samples in each group.

### Biological Assessments *in vivo*

Twenty-seven male Wistar rats (6 weeks of age) were treated in strict accordance with the Guidelines for Animal Experiments of Zhejiang University. Briefly, under general anesthesia induced by an intraperitoneal injection of pentobarbital sodium, the right maxillary first molar was extracted, and a pure-titanium implant (Zhejiang Guangci Medical Appliance Company, Ningbo, China) was screwed into the cavity simultaneously. After surgery, all rats were given a powdered diet. At 2 and 4 weeks after implantation, under general anesthesia, the rats were perfused with specific fixative containing 0.05% glutaraldehyde, 4% paraformaldehyde and 0.2% picric acid in 0.1 M of PBS (pH7.4). The maxillae were harvested and immersed in the same fixative for 2 h at 4°C, and transferred into a decalcifying solution containing 5% EDTA and 4% sucrose in 0.01 M of PBS (pH 7.4) for 5-day at 4°C. On the 5th day, the samples in the decalcifying solution were stirred gently on the magnetic stirrers at 4°C, and then the soft tissue surrounding the implant or tooth was carefully separated from the bone, implant, or tooth. The samples were quickly frozen in liquid nitrogen and kept at −80°C. After being balanced at −20°C, soft tissue samples were embedded in O.C.T. compound (Sakura, Tokyo, Japan), and then cut into 10-μm bucco-palatal sections.

#### Hematoxylin and Eosin Staining and Immunofluorescence Analysis

After being rinsed with 0.01 M PBS, part of the cryosections at 2 and 4 weeks after the surgery were stained with hematoxylin and eosin (HE). Then the slices were observed under a light microscope.

The other sections were stained using the immunofluorescence technique. Antibody to laminin α3 (Santa Cruz, United States) and antibody to integrin β4 (Abcam, United States) were used as the primary antibodies. After being rinsed with PBS, the tissue sections were incubated with the antibody at 4°C overnight. Several sections treated with the anti-laminin α3 rabbit antibody were incubated with a goat-anti-rabbit secondary antibody with rhodamine (Jackson ImmunoResearch Labs, United States), and other sections treated with the anti-integrin β4 mouse antibody were incubated with a goat-anti-mouse secondary antibody with Alexa 488 (Invitrogen Corp., United States). The sections were then stained with Hoechst 33258 DNA dye. Finally, the samples were observed and photographed with a fluorescence microscope.

#### Interface Detected by Transmission Electron Microscope

After the soft tissue was harvested, the samples were treated in 2.5% glutaraldehyde at 4°C overnight. Briefly, the samples were postfixed in 1% osmium tetroxide at 4°C for 1 h and stained with 4% uranyl acetate. After being dehydrated six times in graded ethanol and acetone, the samples were embedded in epoxy resin and cut bucco-palatally. Sections were about 100 nm in thickness and then observed on a transmission electron microscope (TECNAI-10, Philip, Netherlands).

#### Detection of Horseradish Peroxidase Penetration

At 4 weeks after implantation, type VI horseradish peroxidase (HRP) (Sigma, St. Louis, United States) was applied topically to nine rats (three rats for each model). The procedure for the topical application of HRP was similar to the previously reported method (Ikeda et al., 2002). Under general anesthesia, cotton floss was immersed in 10 μl of HRP solution, and then laid on the gingival margin surrounding the implant cervical region without imposing any mechanical stress. Then, 10 μl of the same solution was dripped onto the floss every 10 min for 1 h. Similarly, the procedure above was performed in the gingivae around the maxillary left first molar tooth as a control. The preparation of the gingiva section was described above.

### Statistical Evaluation

Data are expressed as the means ± standard deviation (SD). The normality of the data was identified and the homogeneity test of variance was carried out, followed by analysis of variance (ANOVA) to detect differences in all groups. Significant differences in multiple comparisons were examined by the least significant difference (LSD) method. Values of *P* < 0.05 were considered statistically significant.

## Results

### Surface Morphology and Characteristics

**FIGURE 1:**
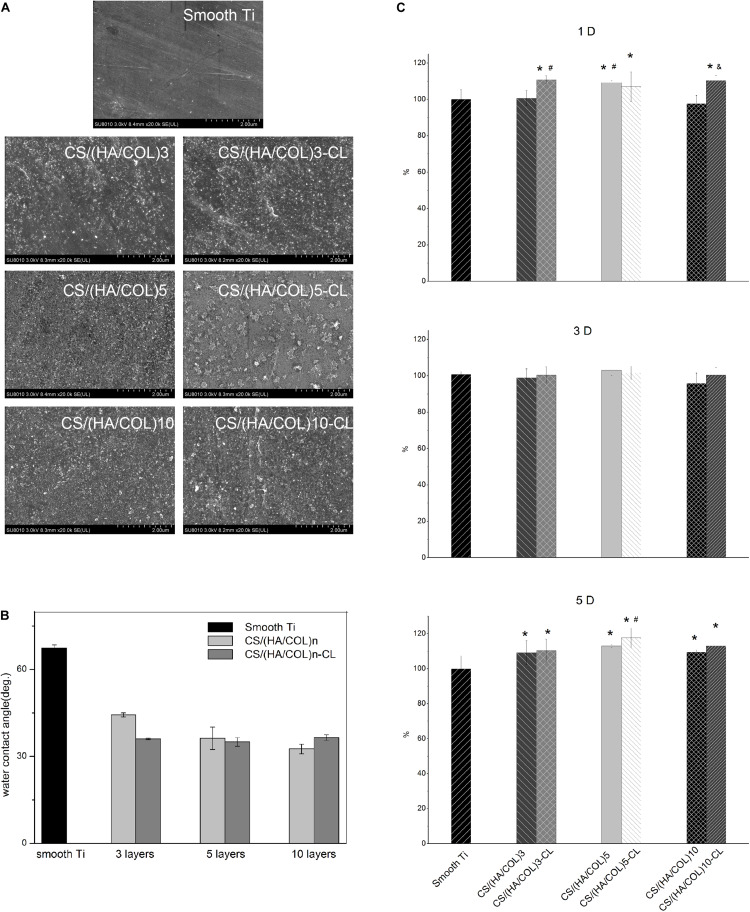
Surface morphology and characteristics of the smooth titanium surface, CS/(HA/COL)n multilayers before and after cross-linking process. **(A)** FSEM scanning results of the different samples, at 20,000× magnification. **(B)** Contact angles of the smooth titanium plate, CS/(HA/COL)n and CS/(HA/COL)n-CL coatings. **(C)** Cell proliferation on different groups for 1, 3, and 5-day. 1-day: ^∗^*p* < 0.05 vs the relative cell viability of HaCaT on the smooth Ti; ^#^*p* < 0.05 vs the relative cell viability of HaCaT on the CS/(HA/COL)3 coating; ^&^*p* < 0.05 vs the relative cell viability of HaCaT on the CS/(HA/COL)10 coating. 5-day: ^∗^*p* < 0.05 vs the relative cell viability of HaCaT on the smooth Ti; ^#^*p* < 0.05 vs the relative cell viability of HaCaT on the CS/(HA/COL)3-CL coating.

The surface morphologies of a polished titanium surface and a titanium plate fabricated with CS/(HA/COL)3, CS/(HA/COL)3-CL, CS/(HA/COL)5, CS/(HA/COL)5-CL, CS/(HA/COL)10, CS/(HA/COL)10-CL are shown in Figure 1A. Parallel grooves were observed on the surface of the polished titanium plate, which was generated during the grinding process. After the assembly of the CS/(HA/COL)n multilayer, the granular-like structures could be seen. As the layers increased, the coating became denser. Compared with the uncrosslinked groups, the granules gathered into a mass in the CS/(HA/COL)n-CL groups.

As shown in Figure 1B, the contact angle of the polished titanium surface was 67.4 ± 1.1°. As the number of the layers increased in the CS/(HA/COL)n group, the contact angle decreased to 44.4 ± 0.7°, 36.3 ± 3.8°, and 32.6 ± 1.6°, respectively. Similarly, the contact angles of the CS/(HA/COL)n-CL coatings decreased and fluctuated around 36° after cross-linking. It indicated that the CS/(HA/COL)n coatings with or without cross-linking could improve the wettability of the polished titanium surface.

### Cell Proliferation Activity

As shown in Figure 1C, the proliferative activity of the HaCaT cells on each group after 1, 3, and 5-day was determined. On the first day, the cell proliferative activities in groups CS/(HA/COL)3-CL, CS/(HA/COL)5-CL, and CS/(HA/COL)10-CL were significantly higher than that in the polished titanium group (*p* < 0.05). The cell proliferative activity in the seven groups appeared to have no significant difference after 3-day of cell culture. On the 5th day, the six coating groups showed significantly higher cell proliferative activity than the polished titanium group (*p* < 0.05), and the cell proliferative activity in CS/(HA/COL)5-CL group was higher than that in CS/(HA/COL)3-CLgroup (*p* < 0.05).

### Condition Optimization

**FIGURE 2:**
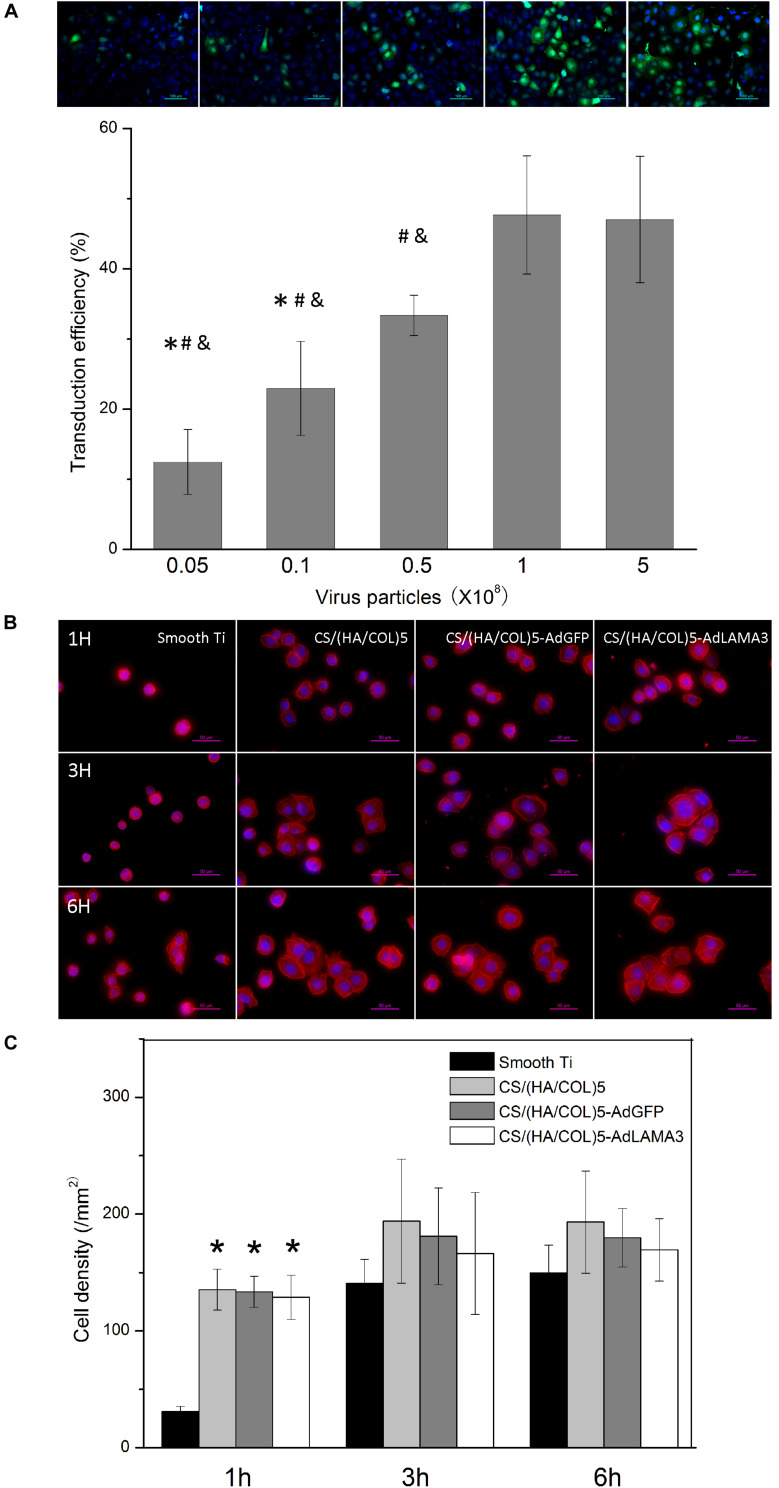
Condition optimization of the coating fabrication and the cell adhesion assessment. **(A)** Optimization of the dose of adenovirus immobilized on the coating according to the cell transfection efficiency. ^∗^*p* < 0.05 vs the cell transfection efficiency of 0.5 × 10^8^ PFU group; ^#^*p* < 0.05 vs the cell transfection efficiency of 1 × 10^8^ PFU group; ^&^*p* < 0.05 vs the cell transfection efficiency of 5 × 10^8^ PFU group. **(B)** The fluorescence microscopic photos showing cellular morphology at the early stage. Original magnification: 400×. **(C)** The cell density on different samples. ^∗^*p* < 0.05 vs the cell density of HaCaT on the smooth Ti.

Based on the above results, the CS/(HA/COL)5 group with five layers was adopted to fabricate the AdLAMA3 immobilized multilayer coating on titanium surface. We further assessed the amount of adenovirus with the optimum transfection rate. Results shown in Figure 2A indicated that transfection efficiency reached the peak and achieve stationary phase (nearly 48%) when the dosage of adenovirus was 1 × 10^8^ pfu.

### Cell Adhesion Assessment

Cell adhesion in the early stage was evaluated. As shown in Figures 2B,C, the CS/(HA/COL)5, CS/(HA/COL)5-AdGFP, and CS/(HA/COL)5-AdLAMA3 groups displayed a significantly higher intensity of the attached cells after seeding for 1 h (*p* < 0.01). After 3 h, the HaCaT cells spread mostly on the three coating groups, while the cells were dotted without the cytoskeleton spreading on the polished titanium surface. These results indicated that the coatings fabricated by the LBL technique improved the cytocompatibility of the native titanium surface, while there was no difference among the three coating groups.

### Histological Observation

**FIGURE 3:**
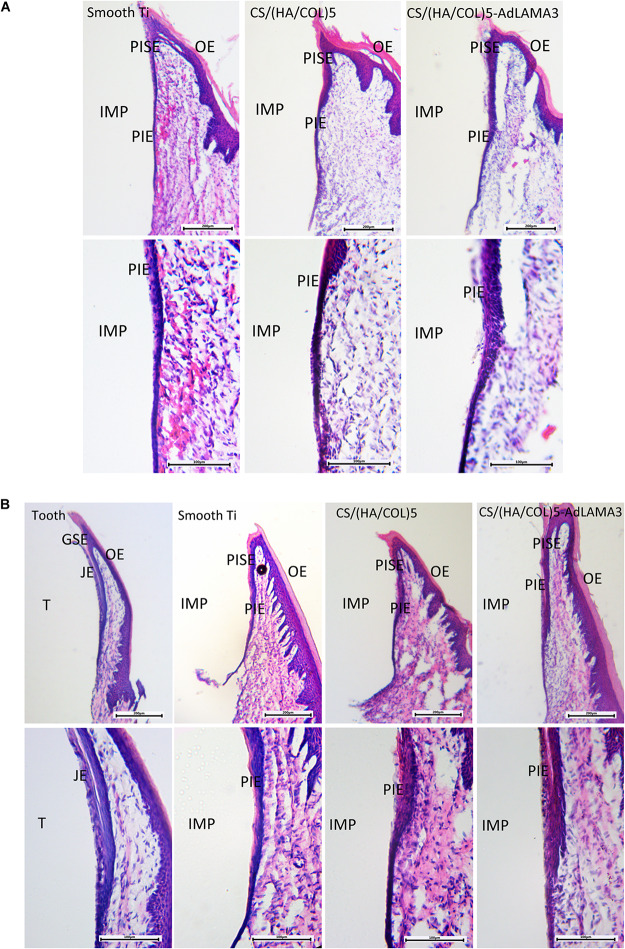
Formation of the peri-implant epithelium 2 weeks **(A)** and 4 weeks **(B)** after surgery in Wistar rats. Magnification: 40× (upper) and 100× (lower). IMP, implant; PISE, peri-implant sulcular epithelium; PIE, peri-implant epithelium; T, tooth; GSE, gingival sulcular epithelium; JE, junctional epithelium; OE, oral epithelium.

The gingival epithelium around the natural tooth consists of the gingival sulcular epithelium (GSE) and the junctional epithelium (JE). Similarly, the gingival epithelium around the implant consists of the peri-implant sulcular epithelium (PISE) and PIE. As shown in Figure 3A, a thin PIE formed around the implants after 2 weeks without remarkable soft tissue inflammation and no cells with the morphology of the junctional epithelial cells were observed, which coincided with Shioya’s study (Shioya et al., 2009). There was a free epithelium band on the upper 1/3 interface between the tooth and the gingival epithelium. However, 4 weeks after implantation, a part of the PIE was lacking at the coronal side of the interface of the smooth implant group and the CS/(HA/COL)5 coating group (Figure 3B). Beginning at the tip of the PISE and extending toward the coronal side, a short and thin layer of the epithelium formed along the surface of the implants modified with CS/(HA/COL)5-LAMA3. Moreover, the epithelial cells covering the CS/(HA/COL)5-LAMA3 coated implant surface was almost identical to the cells of the JE around the natural tooth, of which the intercellular space was larger than the other cells in the junctional epithelium.

**FIGURE 4:**
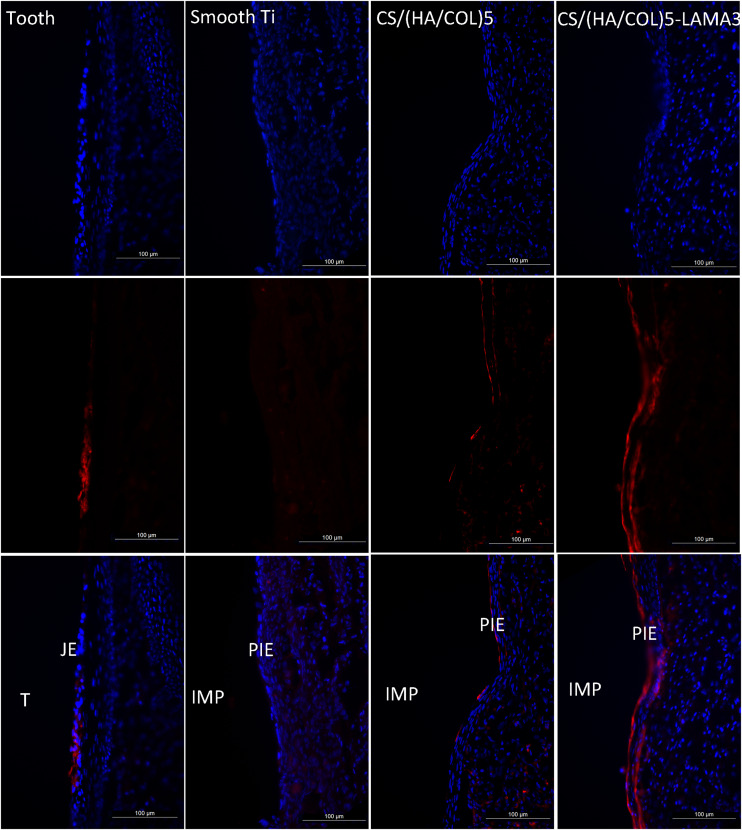
Immunofluorescent microscopy expression of laminin α3 in the junctional epithelium and the peri-implant epithelium along the implant surface 2 weeks after surgery in Wistar rats: laminin α3 (red) and nuclei (blue). Samples were examined under 200× magnification. IMP, implant; PIE, peri-implant epithelium; T, tooth; JE, junctional epithelium.

**FIGURE 5:**
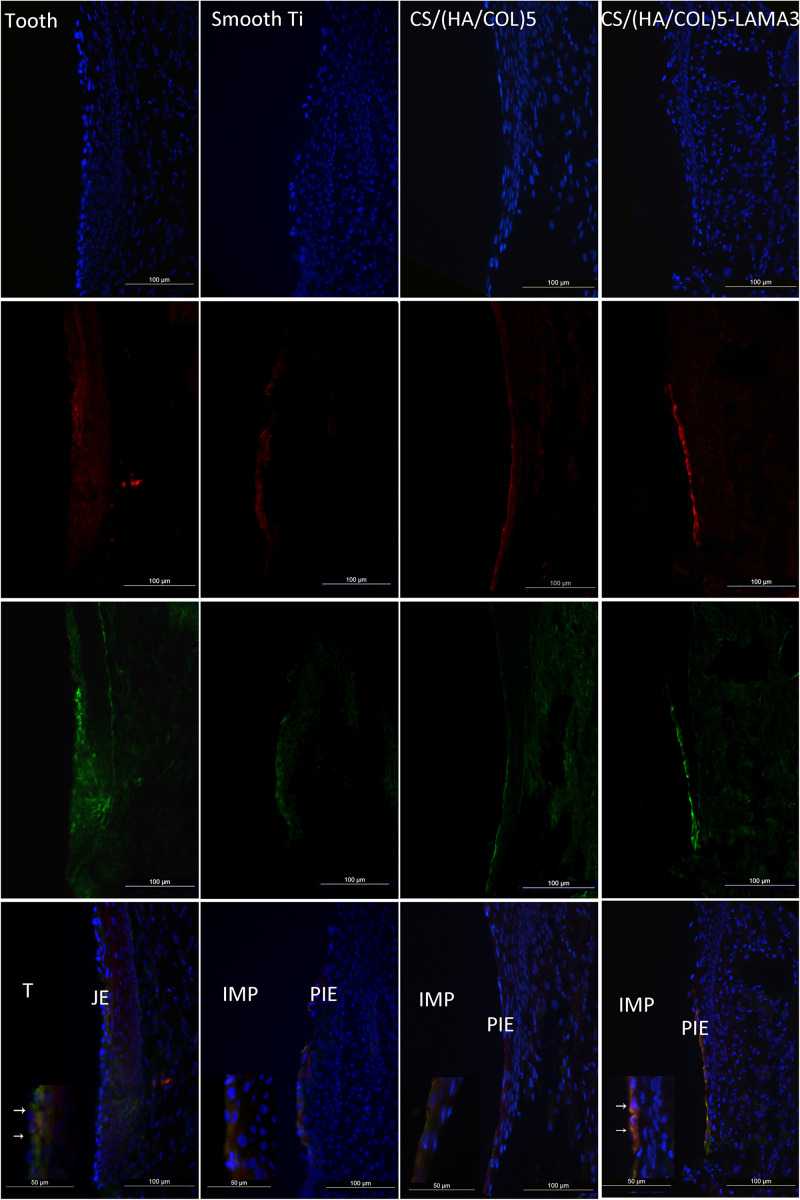
Immunofluorescent microscopy expression of laminin α3 and integrin β4 in the junctional epithelium and the peri-implant epithelium along the implant surface 4 weeks after surgery in Wistar rats: laminin α3 (red); integrin β4 (green); and nuclei (blue). Samples were examined under 200× and 400× magnification (detailed view in the lower-left corner). IMP, implant; PIE, peri-implant epithelium; T, tooth; JE, junctional epithelium.

From the results of the immunofluorescence assay at 2 weeks after surgery (Figure 4), the immunostaining of laminin α3 along the implant-PIE interface and the PIE-connective tissue interface was obviously observed in the group of CS/(HA/COL)5-LAMA3. Both of the laminin α3 positive bands extended from the coronal side to the apical side, which was consistent with the results reported previously (Atsuta et al., 2012). At 4 weeks after surgery, the intensity of laminin α3 in the implant groups was weaker, which indicated that the expression of laminin α3 mainly happened at the early stage of re-epithelization. The co-localization of integrin β4 and laminin α3 was observed in the middle 1/3 of the interface of tooth-JE and the lower 2/3 of the interface of implant-PIE (Figure 5).

### Transmission Electron Microscopy Observation

**FIGURE 6:**
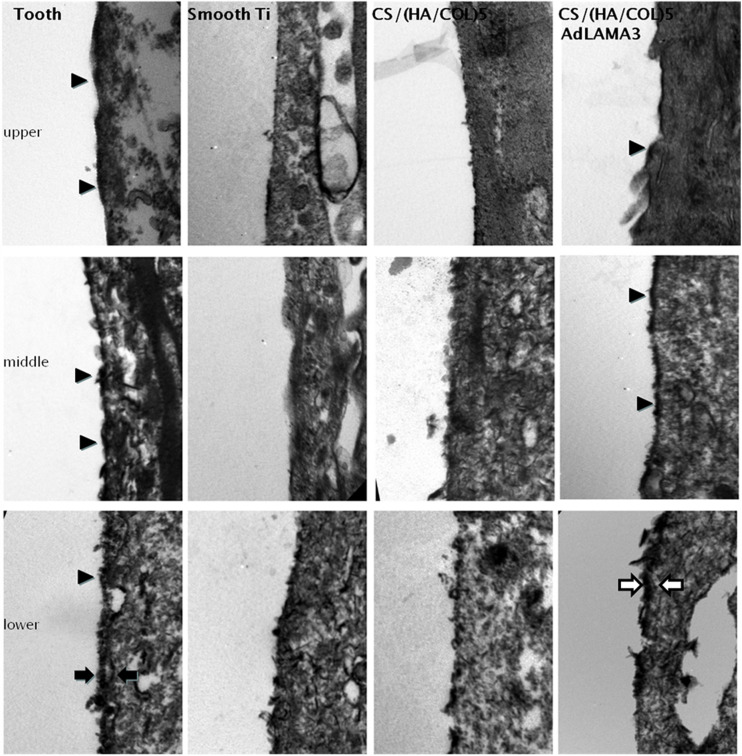
The interface between the tooth/implant and the gingival epithelium was detected by TEM 4 weeks after surgery in Wistar rats: HDs (indicated by the black arrowhead), IBL (indicated by the black arrow), and IBL precursor (indicated by the white arrow). Magnification: 20,500×.

With the transmission electron microscopy (TEM) observation (Figure 6), many continuous hemidesmosomes were observed on the enamel side of the JE and the IBL, including the lamina densa and lamina lucida, appearing at the lower 1/3 of the interface. The interface of the cells and the implants was relatively straight and there were several electron-dense patches observed at the upper and middle interface in the CS/(HA/COL)5-LAMA3 group, which might be the hemidesmosome precursor. In addition, there was an electron dense band along the lower 1/3 of the interface. In reference to the results of the immunofluorescence assay, the electron-dense band might be an extracellular matrix containing laminin332, that is, the precursor of the IBL. However, typical HDs and the IBL or the electron-dense band were not present in the smooth titanium implant and the CS/(HA/COL)5 coating groups.

### Light Microscopy of HRP Penetration Into the Soft Tissue

**FIGURE 7:**
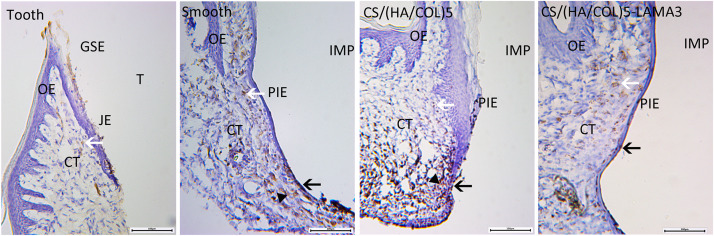
Light microscopy of horseradish peroxidase penetration into the soft tissues around the tooth and experimental implants. In all the groups, the DAB reaction with the endogenous peroxidase was observed (white arrow). In the CS/(HA/COL)5-LAMA3 group, the DAB reaction based on HRP (black arrow) was restricted to the PIE cell layers. In the smooth and CS/(HA/COL)5 groups, the HRP reaction (black triangle) penetrated through the connective tissue throughout the epithelial layer in the apical region of the PIE. Magnification: 100×. IMP, implant; PIE, peri-implant epithelium; T, tooth; JE, junctional epithelium; OE, oral epithelium; CT, connective tissue.

In the group with the natural teeth, the HRP reaction was weak in the intercellular spaces between the JE cells and in JE cells (Figure 7). DAB-positive reaction based on endogenous peroxidase could be detected in all the groups. In the implant groups, HRP-positive vesicles and granules was not only found in the epithelial layers, but also in the connective tissue in the deep and inner layers of the soft tissue. Especially in the smooth titanium group and the CS/(HA/COL)5 coating group, a strong HRP reaction was seen in the intercellular spaces and throughout the cytoplasm of the PIE cells and the connective tissues in the apical region. Compared with those of the other two implant groups, the HRP reaction was limited to the outermost and the innermost cell layers of the PIE in the CS/(HA/COL)5-LAMA3 group.

## Discussion

The defense between the PIE and implant interface is much weaker than that between the JE and tooth, possibly owing to a lack of adhesion structures (HDs and IBL) at the PIE–implant interface. Although many surface modifications have been studied for promoting the biological epithelial sealing at the implant surface in the cervical region, such as hydrofluoric acid–modified surface (Pham et al., 2019), quercitrin-nanocoated titanium surfaces (Gomez-Florit et al., 2016) and CaCl_2_ hydrothermal treatment of titanium implant surfaces (Oshiro et al., 2015). In the study, LAMA3 gene delivery method was used to promote the expression of laminin α3 in epithelial cells and the integration of HDs on the interface for improving the biological sealing between the implant and the gingival epithelium. The novelty of the research is that we immobilized anti-hexon IgG on the multilayer coating and meanwhile, combined the adenoviral vector with anti-hexon IgG through the specificity between antigen and antibody. Adenoviral vectors were restricted to the titanium surface compared to physical immersion or non-specific combination. The results demonstrated that the AdLAMA3 gene transfer promotes early attachment of epithelial cells and re-epithelization of PIE.

Laminin332 is a component of the IBL, and is important in promoting the adhesion of epithelial cells onto the tooth/implant surface. Atsuta et al. reported that laminin332 induces cell migration during PIE formation after Ti implant placement in rats (Atsuta et al., 2005a, b). Wang et al. (2019) indicated that the biological sealing around the transmucosal sites of implant could be promoted by the release of plasmid pLAMA3-CM (the C-terminal globular domain of LAMA3) from a titanium surface. These studies also showed that the leading edge of mucosal wound of sulcular epithelium and the underlying connective tissue express laminin332 during wound healing.

The sequence divergence within the G domain of α3 chain of laminin332 is functionally distinct from other laminin (Ryan et al., 1994). The G domain located at the C-terminal end of the α3 chain contains five repeating LG domains (LG1-5), among which, LG1-3 interacts with the α3β1, α6β1, and α6β4 integrins, while LG4 and LG5 combine with syndecan-1 and -4 (Rousselle et al., 2019). Liu et al. (2019) successfully used Ti modification with chimeric peptideschimeric peptides (derived from the LG3 domain of the α3 chain of laminin332) to improve epithelial sealing on Ti surfaces with the potential to prevent peri-mucositis and peri-implantitis. In the study, the G domain of α3 chain of laminin332 was selected as the functional section, which plays an important role in the formation of HDs. The upregulation of the G domain of laminin α3 has the potential to promote the formation of HDs at the implant-PIE interface. Moreover, the G4/5 domain in the α3 chain could facilitate the deposition of laminin332 into the provisional basement membrane in epidermal wounds (Sigle et al., 2004). Thus, the overexpression of the laminin α3 chain may accelerate the process of re-epithelization surrounding the implant.

In previous research, a multilayer gene coating was fabricated with plasmid cDNA via the LBL assembly (Yang et al., 2016). As we know, the surface composition and surface roughness of an implant play significant roles in protein adsorption and cellular behavior (Zhang et al., 2017). Compared with the polished titanium surface, the hydrophilia and the cytocompatibility of the multilayer coating before and after cross-linking became better. Despite that the non-viral gene therapy is promising, it presents greater challenges with regard to gene-transfer efficiency. Among all currently available viral vectors, adenovirus vector is one of the most efficient gene delivery system (Lee et al., 2017). The challenges of adenovirus vector include reactions with the immune system, viral longevity and vector packaging capacity. However, the major obstacle is the lack of appropriate targeting of viral vectors, which allows only partial exploitation of the great potential of gene therapy (Waehler et al., 2007). In a successful gene therapy, the target genes must be delivered to and expressed in target cells and tissues, without affecting non-target cells (Lee et al., 2017). Therefore, we have established a substrate-mediated gene transduction system to deliver LAMA3 gene to the attached epithelial cells on the titanium surface. Unlike the plasmid cDNA, the adenovirus vectors are not electrically charged. In this study, virus immobilization was performed by the LBL technique and the specific binding of the antibody to antigen. Moreover, the chitosan assembled in the LBL coating could safely increase adenoviral infectivity in mammalian cells, particularly those with poor susceptibility to adenoviral infection (Kawamata et al., 2002). It has been hypothesized that AdLAMA3 localization should enhance local efficacy, reduce the dosage of the viral vector and minimize virus spreading to distant sites, which is decided by antibody immobilization.

In the animal experiment, the rat model with the extraction of the first upper right molar was established. From the morphological observation after 2 weeks, the epithelium layer along the implant surface was longer than the natural junctional epithelium. It has been reported that the regeneration of the implant epithelium appeared during the healing process of long junctional epithelium (Larjava et al., 2011). Listgarten et al. (1982) reported that the appearance of the long junctional epithelium was just a temporary stage, rather than the final stage of wound healing. After 4 weeks, the epithelium layer at the implant-gingiva interface became thicker and shorter. Especially in the CS/(HA/COL)5-AdLAMA3 group, the epithelium extended toward the coronal part. Uno et al. (1998) reported that the proliferative activity of the long junctional epithelium was maintained because of the basal cells in the connective tissues, and the basal cells stopped migrating directly to the root surface when the process of re-epithelization was accomplished, and then the long junctional epithelium finally desquamated from the surface of the connective tissues. This might be the reason that the PIE was shorter after 4 weeks than after 2 weeks.

During the wound healing process, laminin332 appears to be the first marix component laid down onto the wound bed, and its expression precedes that of all other ECM components (Rousselle et al., 2019). At the leading edge of the wound repair, the molecular profiling in a mice wound model identified laminin and fibronectin as the major ECM proteins expressed by keratinocytes (Aragona et al., 2017). Therefore, laminin332 deposition can be a significant indication of epithelium wound healing and an index of re-epithelization surrounding the implant. After 2 weeks of implantation, we detected that the positive band of laminin332 deposition extended from coronal to apical at the implant-PIE interface, especially in the CS/(HA/COL)5-AdLAMA3 group. Since the expression of laminin332 mainly happened at the early stage of wound healing (Iorio et al., 2015), the expressions of laminin332 at 4 weeks were weaker than those at 2 weeks.

Transmission electron microscopy observation is one of the optimal techniques to detect the structure of HDs. In our research, there were few electron-dense plaques observed at the lower 1/3 of the implant-epithelium interface in the smooth implant group and the CS/(HA/COL)5 group. In the CS/(HA/COL)5-AdLAMA3 group, several continuous HDs were observed along the middle 1/3 interface of the implant-epithelium, and a thick electron-dense band was observed at the lower 1/3 interface, which was probably about to become the former matrix of the internal basal lamina. Consistent with the results of TEM observation, the biological sealing of the CS/(HA/COL)5-AdLAMA3 group was found to be better than those of the other two implant groups with the HRP penetration evaluation.

Based on the above results, the implant surface modified with the CS/(HA/COL)5-AdLAMA3 coating could promote the epithelial attachment and the process of re-epithelization. Laminin332 is an optimum ligand for keratinocyte adhesion and migration, and mediates cell behavior in the process of wound healing. When the JE is damaged, the basal cells in the GSE could differentiate into keratinocyte cells. At the early stage of wound healing, the epithelial cells extended toward the apical portion, and the epithelial cells at the forefront edge of the wound could secrete and deposit the extracellular matrix containing laminin332. After 4 weeks of implantation, the re-epithelization surrounding the implant was almost completed, and laminin332 interacted with integrin α6β4 to promote the integration of HDs at the implant-PIE interface.

However, the formations of IBL and HDs in the group of CS/(HA/COL)5-AdLAMA3 coating were not as perfect as those at the tooth-JE interface. Compared with the cells of JE, the cells of PIE have weak proliferative activity by proliferating cell nuclear antigen detection (PCNA) (Inoue et al., 1997). Furthermore, the formation mechanisms of the PIE and the JE are different. In the process of epithelial cell renewal and reproduction, the basal cell of the GSE has a strong capacity for regeneration and could differentiate into keratinocyte, including the peri-implant epithelial cell. Recent studies indicated that oral implants trigger a mild immune reaction, and survive in the body due to balanced defense reactions based on the individual host immune system (Albrektsson et al., 2019a, b). Hence, we need to do further research to investigate the mechanisms of immunoreaction and re-epithelization around the implant surface. In addition, the perfect cervical surface of the implant should include both the ability to promote the biological sealing (Oshiro et al., 2015) and the potential to reduce bacteria surface colonization (Ferraris et al., 2019). In our previous research, antimicrobial peptide-loaded coatings (AMP) was fabricated on the titanium surface, which could decrease the growth of both a Gram-positive aerobe (Staphylococcus aureus) and a Gram-negative anaerobe (Porphyromonas gingivalis) up to one month (Shi et al., 2015). Thus, how to combine the AdLAMA3 *in situ* transfection system with the antibacterial coating will be an important direction for future researches.

## Conclusion

In conclusion, the titanium surface functionalized with AdLAMA3 was successfully established after condition optimization. The multilayer coating could promote the expression of laminin α3 in epithelial cells, and efficiently improve the formation of the PIE. The next step will be further study on the mechanism of re-epithelization of PIE and the immune response involved in the process.

## Data Availability Statement

All datasets generated for this study are included in the article/supplementary material.

## Ethics Statement

The animal study was reviewed and approved by Animal Experiments of Zhejiang University.

## Author Contributions

GY and JZ conceptualized the study and designed the experiments. JZ drafted the manuscript. JZ and YL conducted the experiments. JL critically revised the manuscript for intellectual content. YS and JH analyzed and interpreted the results. All authors gave permission to the final draft of the manuscript.

## Conflict of Interest

The authors declare that the research was conducted in the absence of any commercial or financial relationships that could be construed as a potential conflict of interest.
